# Conversion of Furfuryl Alcohol into Ethyl Levulinate over Glucose-Derived Carbon-Based Solid Acid in Ethanol

**DOI:** 10.3390/molecules24101881

**Published:** 2019-05-16

**Authors:** Geng Zhao, Ming Liu, Xinkui Xia, Li Li, Bayin Xu

**Affiliations:** 1 Analysis and Testing Center, College of Chemistry and Chemical Engineering, Xinyang Normal University, Xinyang 464000, Henan, China; lmminga@163.com (M.L.); xytclily@163.com (L.L.); burenbayin@163.com (B.X.); 2 College of Food Science, Xinyang Agriculture and Forestry University, Xinyang 464000, Henan, China; xynzxb@126.com

**Keywords:** furfuryl alcohol, ethanol, ethyl levulinate, carbon-based solid acid

## Abstract

In this study, a carbon-based solid acid was created through the sulfonation of carbon obtained from the hydrothermal pretreatment of glucose. Additionally, ethyl levulinate, a viable liquid biofuel, was produced from furfuryl alcohol using the environmentally benign and low-cost catalyst in ethanol. Studies for optimizing the reaction conditions, such as reaction time, temperature, and catalyst loading, were performed. Under the optimal conditions, a maximum ethyl levulinate yield of 67.1% was obtained. The recovered catalyst activity (Ethyl levulinate yield 57.3%) remained high after being used four times, and it was easily regenerated with a simple sulfonation process. Moreover, the catalyst was characterized using FT-IR, XRD, SEM, elemental analysis, and acid-base titration techniques.

## 1. Introduction

Because of the recent diminishment of fossil fuel resources, as well as environmental degradation resulting from greenhouse gas emissions, significant effort has been devoted to converting renewable biomass into liquid fuels, fuel additives, and organic bulk chemicals [1,2,3]. Ethyl levulinate (EL) is considered to be a potential liquid biofuel for the future [4]. Ethyl levulinate is a short chain fatty ester, with properties similar to the fatty acid methyl ester of biodiesel. It has many excellent benefits, such as high lubricity, non-toxicity, flashpoint stability, and good flow properties under cold conditions [5]. Moreover, EL is of particular interest due to its extensive applications in the flavoring, solvent, and plasticizer sectors [6]. Additionally, it has found applications in the area of organic chemistry for the synthesis of the viable biofuel γ-valerolactone [7,8].

Currently, acid-catalyzed esterification of levulinic acid (LA) is the most efficient method for the synthesis of EL, with high yields regularly achieved [9,10,11,12]. However, LA is an expensive raw material, because it needs to be prepared from carbohydrates or biomass firstly, and then needs to be purified for synthesis of ethyl levulinate [13,14]. On the other hand, an increasing number of studies have focused on the direct production of EL from biomass in ethanol, such as hexose [15,16,17], cellulose [18], wood, bagasse [19], and wheat straw [20]. As a direct feedstock, raw biomass is abundant and inexpensive; however, the highest yield (55%) was achieved starting from the aforementioned feedstock (hexose).

**Scheme 1:**
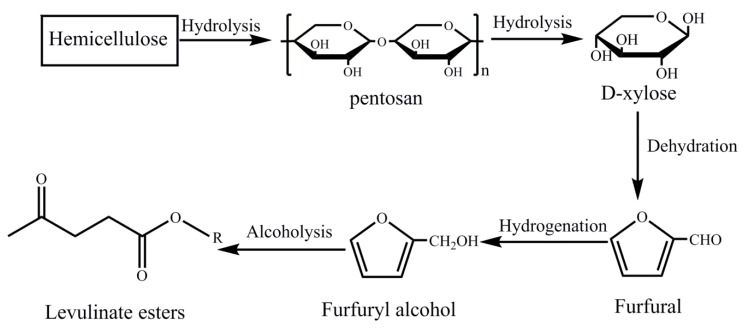
The route of synthesizing levulinate esters from hemicellulose.

As can be seen from Scheme 1, furfuryl alcohol (FA) is produced industrially via the hydrogenation of furfural [21,22]. It should be noted that furfural can be derived from the hydrolysis and dehydration of xylan contained in hemicellulose-rich biomass, including corncobs, corn stock, rice hulls, and olive stones. Presently, there are more than 400 factories producing furfural in China, and the annual global production of furfural exceeds 700,000 tons [23,24]. Therefore, furfural and FA are not fully utilized in the chemical market [25]. The conversion of FA into EL is regarded as an economic and convenient strategy that has several advantages, including lower reaction temperature, cheaper raw material, and higher product yield. However, little effort has been made to employ this approach to produce EL. Some researchers have obtained EL from FA catalyzed by sulfuric acid [24], acidic ion-exchange resins [26], sulfonic acid functionalized ILs [27], and aluminosilicates [28]. It is important to note that these catalysts, except for sulfuric acid, can be extremely expensive due to their complex preparation processes. Furthermore, sulfuric acid and sulfonic acid functionalized ILs are homogeneous, creating serious drawbacks in terms of separation, recycling, and equipment corrosion. Therefore, it is necessary to develop an environmentally benign and low-cost solid acid catalyst for the conversion of FA into EL. In the resulting studies, high EL yields were achieved, but under longer reaction time (24 h) or lower substrate concentration (1.0 wt.%), which may potentially be hurdles in the process of the commercialization of EL.

In this study, an inexpensive and robust carbon-based solid acid catalyst was successfully synthesized and evaluated for the conversion of FA into EL in ethanol. A maximum EL yield of 67.1% was achieved under the optimal conditions, and all the conversion reactions were performed in triplicate. The recovered catalyst possessed good catalytic activity (EL yield 57.3%), even after four cycles, and it was easily regenerated by a simple sulfonation process. Moreover, the catalyst was characterized using FT-IR, XRD, SEM, elemental analysis, and acid-base titration techniques.

## 2. Results and Discussion

### 2.1. Catalyst Characterizations

**Figure 1:**
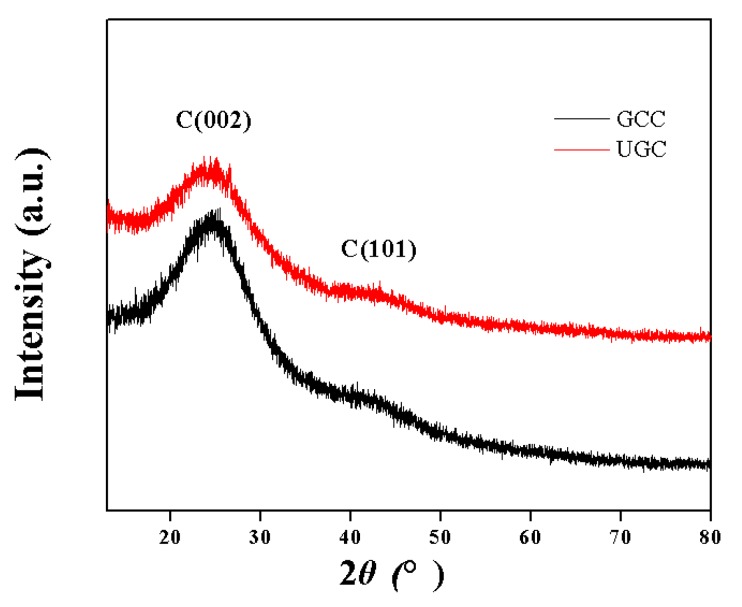
XRD patterns of unsulfonated glucose-derived carbon (UGC) and glucose-derived carbonaceous catalyst (GCC).

Structural information about unsulfonated glucose-derived carbon (UGC) and glucose-derived carbonaceous catalyst (GCC) samples were obtained by FT-IR and XRD. Figure 1 shows the XRD patterns of two samples, which exhibit two 2θ° weak diffraction peaks between 10 and 30° and 35 and 50°, which can be assigned to C (002) and C (101), respectively. The results indicate that amorphous carbon, composed of aromatic carbon sheets, is oriented in a random fashion [29]. Two samples have similar XRD patterns, indicating that the sulfonation process had no effect on the morphology of carbon.

**Figure 2:**
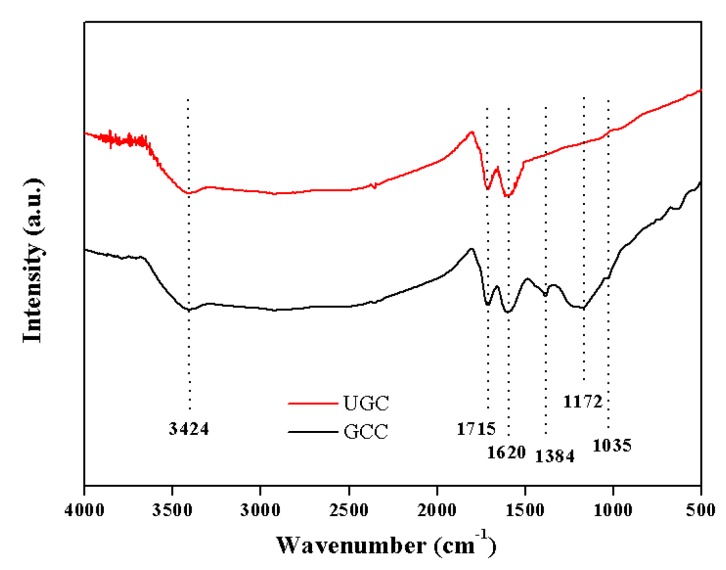
FT-IR spectra of UGC and GCC.

The UGC and GCC were further characterized by FT-IR, and the results are shown in Figure 2. The vibration bands at 1384, 1172, and 1035 cm^−1^ (SO_3_ stretching) on GCC are evidence that -SO_3_H groups were successfully incorporated into the UGC. In addition, the absorption bands at 1715 and 1620 cm^−1^ correspond to -C=O (carbonyl) and -OH (hydroxyl) bending vibrations, implying that carboxyl groups exist on the prepared carbon materials. The bands at 3424 and 1620 cm^−1^ can be assigned to C-OH stretching and -OH bending vibrations, implying that hydroxyl groups exist on the prepared carbon materials [30,31].

### 2.2. Identification of the Product

The mechanism responsible for the conversion of FA to EL is complex. Additionally, it is also possible for FA to react with ethanol at higher temperatures to form ether, poly FA, and other by-products [24,27]. In an attempt to identify the product, the liquid phase of the reaction mixture obtained from the conversion of FA in the presence of GCC (at 423 K for 60 min) was analyzed using GC-MS. The GC-MS spectra are given in Figures S1 and S2, located in the Supplementary Materials section. GC-MS analysis of the reaction mixture shows the presence of EL at a retention time of 10.54 min. The mass spectrum of the product exhibits molecular ionization at *m/z* 144, corresponding to the molecular formula of EL (C_7_H_12_O_3_). Furthermore, the base peak fragmentation of the ester is related to CH_3_CO (*m*/*z* = 43). Two other important ion peaks, located at *m/z* 99 and 129, are due to the formation of the ion acylium RCO^+^, resulting from the loss of either the alkoxy or methyl group from the ester. Additional bond cleavages occurred through some pathways and created fragment ions at 40, 55, 74, and 101 *m/z* [10].

### 2.3. Effect of Reaction Parameters

**Figure 3:**
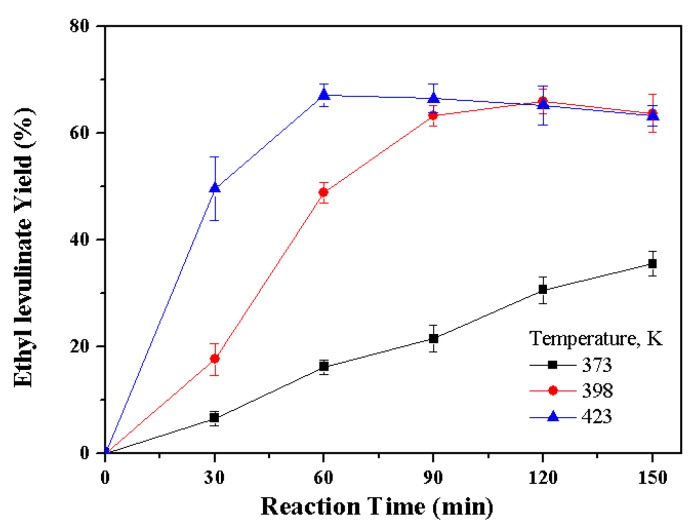
Effect of reaction temperature on EL yield. Reaction conditions: furfuryl alcohol (FA) 1 g, ethanol 19 g, GCC 0.5 g.

The catalytic conversions of FA were conducted with temperatures ranging from 373 to 423 K, using differing time intervals, to deduce the optimum conditions for increasing the EL yield. As seen in Figure 3, the reaction temperature and time have a great influence on the overall EL yield. Under lower temperature condition, only 35.5% EL yield was achieved after the reaction time of 150 min at 373 K. Elevating the reaction temperature to 423 K increased the yield of EL rapidly with the extension of the reaction time, and the equilibrium point (67.1%) was reached by 60 min. Moreover, at 398 and 423 K, when EL yields reached their peak values, longer reaction times resulted in lower yields of EL. The low yield of EL indicates that the substance might decompose to some extent, and more undesired byproducts were formed under higher temperatures [32].

**Figure 4:**
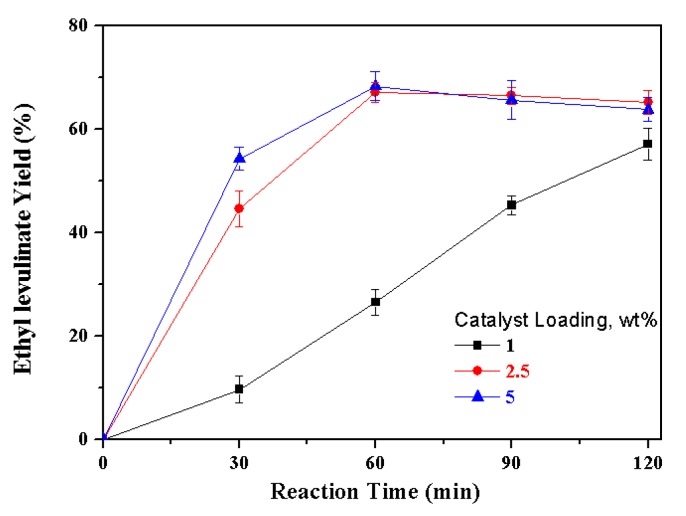
Effect of catalyst dosage on EL yield. Reaction conditions: FA 1 g, ethanol 19 g, temperature 423K.

Subsequently, the effect of the catalyst loading dosage on the conversion of FA into EL was studied, and the results are presented below in Figure 4. When the reaction time was 60 min and 1 wt.% GCC was used, the EL yield was only 26.5%. Increasing the GCC dosage to 2.5 wt.% resulted in a remarkable increase in EL yield to 67.1%. However, when the catalyst loading dosage was doubled to 5.0 wt.%, the yield of EL increased by only 1.3%. Moreover, after 60 min, the EL yield decreased faster when 5.0 wt.% GCC was used, which could be attributed to the increased availability of acid sites, derived from excessive GCC, facilitating the decomposition of EL. Thus, it was determined that a 2.5 wt.% catalyst loading dosage was optimal.

### 2.4. Recyclability of GCC

**Figure 5:**
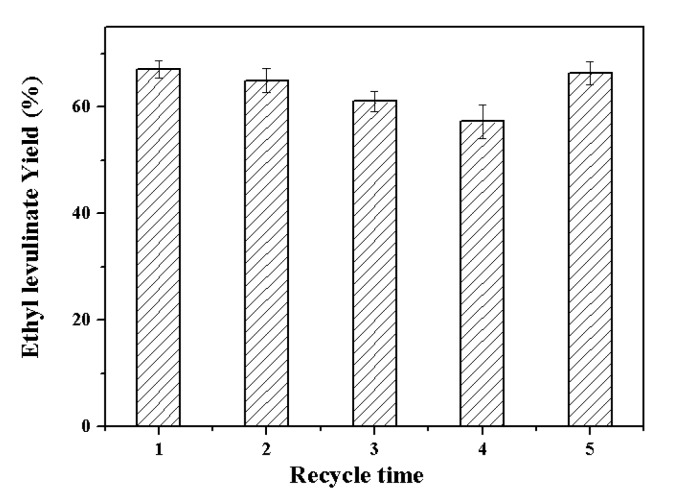
Reuse of the GCC catalyst. Reaction conditions: FA 1 g, ethanol 19 g, GCC 0.5 g temperature 423 K, reaction time 60 min.

The reusability and long-term stability of a given catalyst are extremely important factors in reducing production cost for practical biomass transformation. After the reaction was completed, the GCC was separated from the reaction mixture by filtration, washed 10 times with 10 mL of ethanol, and dried at 378 K for 24 h in a vacuum oven. Then, the GCC was used in the next reaction experiment under the same reaction conditions. As shown in Figure 5, GCC maintained good catalytic activity after four reactions, and an EL yield of 57.3% was still achieved. Subsequently, the four time-recycled GCC was regenerated by simple sulfonation at 453 K for 1 h using a solid/concentrated sulfuric acid ratio of 1:10 under a nitrogen atmosphere. The yield of the regenerated EL was restored to 66.4%, which was comparable to that of the fresh catalyst (67.1%).

Under the same experimental conditions (at 423 K for 60 min), the catalytic activity of UGC (EL yield 11.6%) is much lower than that of GCC (EL yield 67.1%). The remarkable catalytic activity of GCC may be due to the high acidity of the sulfonated carbon catalyst, as well as the presence of -SO_3_H groups on the amorphous carbon catalyst. The acid density and elemental content of the reclaimed GCC are summarized in Table 1. The acid density and the sulfuric content of GCC decreased significantly with the increasing reaction cycles. On the other hand, the carbon content of GCC increased from 52.2 to 60.6 wt.% throughout the course of the four reaction runs. The results indicated that the slight decrease in EL yield was possible due to the leaching of the -SO_3_H group coupled with the deposition of humins on the surface of the GCC studied. In addition, the acid density and elemental content of the regenerated GCC were nearly identical to the fresh catalyst, indicating that the regeneration process not only recovered the catalytic activity of GCC, but also utilized the humins deposited on GCC.

**Figure 6:**
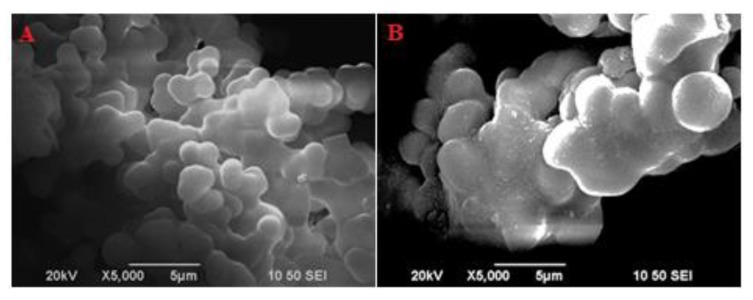
SEM images for fresh catalyst (**A**) and recycled catalyst after four runs (**B**).

The comparative SEM images of the fresh and four time-recycled GCC are shown in Figure 6. It is clear that they both exhibited an intertwined structure and consisted of irregular particles with a size of several micrometers. However, the particles of the four time-recycled GCC were larger, and their surfaces were smoother, indicating substantial humin deposition on the surface of GCC after four reaction runs.

## 3. Materials and Methods

### 3.1. Materials

Ethyl levulinate and furfuryl alcohol were purchased from Aladdin Chemical Reagent (Shanghai, China). Other reagents were obtained from Sinopharm Chemical Reagent (Shanghai, China). All reagents and chemicals were analytical grade and used without further purification or treatment. 

### 3.2. Catalyst Preparation

Using the typical preparation method, glucose (30 g) was dissolved in 50 mL of distilled water in a cylindrical stainless steel pressurized reactor with 100 mL total volume (PARR, Moline, IL, USA). The reactor was sealed and heated to 473 K with agitation rate of 500 rpm for 6 h. After the hydrothermal carbonization process was completed, the solid carbon was separated by filtration, washed with distilled water, and subsequently dried in an oven at 383 K for 24 h. The resulting solid carbon (5 g) was then ground to a powder and heated at 453 K for 5 h in 50 mL of concentrated sulfuric acid (98%) under a nitrogen-rich atmosphere to introduce -SO_3_H groups. The sulfonated mixture was then diluted with 1000 mL of deionized water to form a black precipitate, which was subsequently washed repeatedly in hot distilled water (>353 K) until the sulfate ions were no longer detected in the residual washing water. Finally, the resulting glucose-derived carbonaceous catalyst (GCC) was dried in an oven at 383 K for 24 h.

### 3.3. Catalyst Characterization

The prepared GCC was characterized by Fourier transform infrared spectroscopy (FT-IR), X-ray diffraction spectroscopy (XRD), scanning electron microscopy (SEM), elemental analysis, and acid-base titration. The FT-IR spectrum was recorded over the range of 400 to 4000 cm^−1^ on a Nicolet iS 50 FT-IR spectrometer, made by Thermo Fisher Scientific, Waltham, MA, USA. The XRD pattern of the GCC was recorded by an X’ Pert PRO powder XRD spectrometer (Panalytical, Almelo, The Netherlands) using a Cu Kα radiation source (λ = 0.154 nm). The morphology of the GCC was observed using a S-4800 scanning electron microscope (Hitachi, Tokyo, Japan) at 30 kV. The elemental content of the GCC sample was determined using a Vario EL III elemental analyzer, made by Elementar, Hanau, Germany. The acid density of the GCC was determined via acid-base titration. 

### 3.4. General Procedure for the Synthesis of EL

The synthesis of EL from FA were conducted in a 50-mL cylindrical, stainless steel, pressurized reactor (PARR, Moline, IL, USA). The reactor was heated in an adjustable electric stove. In a typical experiment, FA (1 g), ethanol (19 g), and a given amount of GCC were introduced into the reactor. After the reactor was sealed, the above mixture was heated to the desired temperature and stirred magnetically at 500 rpm. At the end of the reaction, the resulting mixture was cooled to room temperature. The liquid products and solid acid catalyst were separated through centrifugation at 10,000 rpm for 3 min. After the process was completed, the contents were analyzed. 

### 3.5. Analysis of Products

The qualitative analysis of the sample was conducted with a Shimadzu 2010A gas chromatograph (GC) system, coupled with a Shimadzu QP2010 mass spectrometer (GC-MS). The amount of product was analyzed by GC on an Agilent 7890 instrument, equipped with a HP-5 capillary column and a flame ionization detector (FID). The injection port temperature was 523 K, and the detector temperature was 543 K. The column temperature was maintained at 313 K for 4 min, then raised to 523 K with a ramp rate of 15 K/min. The amount of EL present at the end of the process was calculated using an external standard. The yield of EL on a molar basis was calculated as follows:

(1) $$\mathrm{EL}\;\mathrm{yield}\left(\%\right)=\frac{\mathrm{EL}\;\left(\mathrm{mol}\right)}{\mathrm{FA}\;\left(\mathrm{mol}\right)}\times 100\%$$

## 4. Conclusions

An environmentally benign and inexpensive solid acid catalyst GCC was demonstrated to be highly active for use in the conversion of FA into EL in ethanol. A good EL yield of 67.1% was obtained under moderate temperature and short reaction times (423 K, 60 min). GCC could be recycled four times, with little deactivation, and could be easily regenerated with a simple sulfonation process. 

## Figures and Tables

**Table 1:** Total acid density and elemental content of the reclaimed GCC.

Run	Total Acid Density (mmol·g^−1^) ^a^	Element Content (wt.%) ^b^	
		Carbon	Sulfur
1	2.6	52.2	1.5
2	1.9	57.9	1.1
3	1.7	59.2	1.0
4	1.4	60.6	0.9
5 ^c^	2.5	53.1	1.5

^a^ Determined by acid-base titration. ^b^ Obtained by elemental analysis. ^c^ The regenerated GCC.

## Supplementary Materials

The following are available online. Figure S1: GC spectra of the product of the conversion of FA into EL., Figure S2: MS spectra of the product of the conversion of FA into EL.
