# Supplementary material for: Conversion of Furfuryl Alcohol into Ethyl Levulinate over Glucose-Derived Carbon-Based Solid Acid in Ethanol
Source: Molecules. 2019 May 16;24(10):1881. doi: 10.3390/molecules24101881 (PMC6572322; doi:10.3390/molecules24101881)
Supplement: Supplementary file 1 [file molecules-24-01881-s001.pdf]

Supplementary Materials

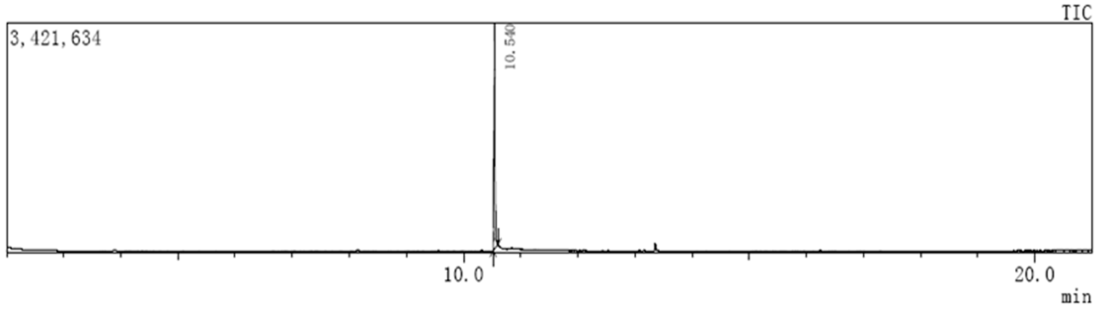

Figure S1. GC spectra of the product of the conversion of FA into EL.

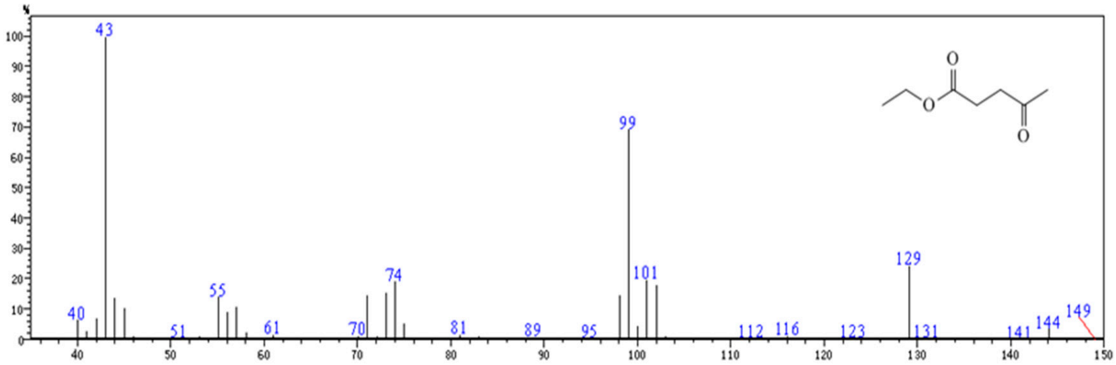

Figure S2. MS spectra of the product of the conversion of FA into EL
